# Application of photoelectrochemical oxidation of wastewater used in the cooling tower water and its influence on microbial corrosion

**DOI:** 10.3389/fmicb.2024.1297721

**Published:** 2024-03-13

**Authors:** Seenivasan Kokilaramani, Alagersamy Satheeshkumar, M. S. Nandini, Jayaraman Narenkumar, Mohamad S. AlSalhi, Sandhanasamy Devanesan, Prabhu Manickam Natarajan, Rajaram Rajamohan, Aruliah Rajasekar, Tabarak Malik

**Affiliations:** ^1^Environmental Molecular Microbiology Research Laboratory, Department of Biotechnology, Thiruvalluvar University, Vellore, Tamilnadu, India; ^2^Department of Microbiology, Sree Balaji Medical College and Hospital, Chennai, Tamil Nadu, India; ^3^Sree Balaji Dental College and Hospital, BIHER University, Chennai, Tamil Nadu, India; ^4^Department of Environmental and Water Resources Engineering, School of Civil Engineering, Vellore Institute of Technology, Vellore, Tamil Nadu, India; ^5^Department of Physics and Astronomy, College of Science, King Saud University, Riyadh, Saudi Arabia; ^6^Department of Clinical Sciences, Center of Medical and Bio-Allied Health Sciences and Research, College of Dentistry, Ajman University, Ajman, United Arab Emirates; ^7^Organic Materials Synthesis Lab, School of Chemical Engineering, Yeungnam University, Gyeongsan-si, Republic of Korea; ^8^Adjunct Faculty, Department of Prothodontics, Saveetha Dental College and Hospital, Chennai, Tamil Nadu, India; ^9^Department of Biomedical Sciences, Institute of Health, Jimma University, Jimma, Ethiopia

**Keywords:** electro oxidation, photo-oxidation, photoelectrochemical oxidation, microbial corrosion, biofilm, cooling tower wastewater

## Abstract

**Background:**

Cooling towers are specialized heat exchanger devices in which air and water interact closely to cool the water's temperature. However, the cooling water contains organic nutrients that can cause microbial corrosion (MC) on the metal surfaces of the tower. This research explores the combined wastewater treatment approach using electrochemical-oxidation (EO), photo-oxidation (PO), and photoelectrochemical oxidation (PEO) to contain pollutants and prevent MC.

**Methods:**

The study employed electro-oxidation, a process involving direct current (DC) power supply, to degrade wastewater. MC studies were conducted using weight loss assessments, scanning electron microscopy (SEM), and x-ray diffraction (XRD).

**Results:**

After wastewater is subjected to electro-oxidation for 4 h, a notable decrease in pollutants was observed, with degradation efficiencies of 71, 75, and 96%, respectively. In the wastewater treated by PEO, microbial growth is restricted as the chemical oxygen demand decreases.

**Discussion:**

A metagenomics study revealed that bacteria present in the cooling tower water consists of 12% of *Nitrospira* genus and 22% of *Fusobacterium* genus. Conclusively, PEO serves as an effective method for treating wastewater, inhibiting microbial growth, degrading pollutants, and protecting metal from biocorrosion.

## Introduction

Cooling towers serve as specialized heat exchangers in which air and water interact closely to reduce the water temperature (Manh et al., 2019). The operational principle involves the passage of water through a heat exchanger, which absorbs heat and spreads it over the tower's top. The interaction with airflow causes the hot water to evaporate, increasing the air's temperature and relative humidity through heat transfer from the water stream to the air stream (Ortíz-Sánchez et al., 2023). This process results in a stream of cold water when the warmed air is released into the environment. The cycle is then repeated by pumping the cooled water from the basin through another heat transfer process (Abdulla et al., 2023). Cooling towers, often referred to as recirculating water systems, play a crucial role in removing excess heat and maintaining temperatures for industrial equipment, including heating, ventilation, and air conditioning (HVAC) systems. The key components of a cooling system include a recirculating pump, a heat exchanger, and a cooling tower (Fitch et al., 2022). Maintenance is a critical aspect of cooling towers. Poor maintenance leads to the accumulation of dust particles, microbes, debris, and other particles on cooling tower components. The accumulation of these particles can block airflow, constraining the operation of fans and pumps. As a consequence, cooling towers have a decreased lifespan and suffer from inefficiencies and loss of cooling efficacy, leading to economic losses and unreliable operations (Martínez-Huitle and Panizza, 2018). The uncontrolled growth of microbes that alter the chemical parameters of the cooling tower systems pose a significant challenge to the industry. Biofilms dominate cooling water systems, promoting micro-biofouling, which can obstruct the operations of these systems (Bhandari and Ranade, 2014; Reddy and Osborne, 2020).

Microbial corrosion occurs between metal surfaces and microbes present in wastewater (aqueous solution), with microbial growth, which depends on the volume of nutrients derived from organic matter, serving as a carbon source (Abdulla et al., 2023; Parthipan et al., 2023). Microbes adhere to exposed surfaces, leading to the production of biopolymers, primarily extracellular polymeric substances (EPS), forming biofilm layers. The subsequent oxidation and reduction processes occurring on metals result in the formation of pitting corrosion (Elumalai et al., 2021). Additionally, salts and organic compounds in the cooling tower water contribute to high salt concentrations and deposition, affecting its overall functioning (Saha et al., 2020). The parameters of cooling tower water, including calcium ions, total hardness, alkalinity, iron, phosphorus, orthophosphate, chloride, sulfur dioxide, conductance, turbidity, and chemical oxygen demand (COD), are analyzed and maintained to proper concentrations (Bustos-Terrones et al., 2021). Wastewater, characterized by a high concentration of inorganic compounds, induces heavy corrosion due to ion exchanges between compounds and metals (Guiamet and Gómez De Saravia, 2005). Industrial sectors frequently use ozonation and chlorination, among other disinfection processes, to treat and recycle wastewater; nevertheless, these systems have downsides, including high maintenance costs and operational concerns (Lazarova et al., 1999). Adsorption and electrochemistry are two removal methods that have been developed recently to clean wastewater (Fomina and Gadd, 2014; Reddy and Osborne, 2022). Wastewater is often subjected to mechanical biotreatment to remove hazardous substances and germs. Microbiological pollutants are assessed and tracked in relation to quality criteria (Musa et al., 2010). Photo-oxidation (PO), electrochemical oxidation (EO), and photoelectrochemical oxidation (PEO) are employed in industrial wastewater treatment due to their strong oxidation capacities to reduce contaminants (Zheng et al., 2022). There are primarily two approaches to electro-oxidation (EO). One is direct oxidation, which occurs on the electrode surface, and it can be stopped by the electric field effect, hydroxyl radicals (OH), hydrogen peroxide, ozone, or hydrogen radicals (Anadebe et al., 2020). The formation of various aqueous solution oxidants, such as sulfate or chlorine species, is the foundation of the second strategy, known as indirect oxidation (Sierra-Sánchez et al., 2022). When applied correctly, the efficient processes of anodic oxidation (EO) and ultraviolet radiation (PO) can both reduce and eliminate pollutants found in wastewater (Ungureanu et al., 2020). The EO process relies on the production of potent oxidants, such as hydroxyl radicals and oxide radicals (${\text{O}}_{2}^{-}$), which react with contaminants to accelerate the degradation mechanisms (Cotillas et al., 2016). The straightforward method of PO relies on irradiating semiconductors with light to excite higher electron states (e^−^), which move from the valence band to the conduction band and leave holes (“h^+^”) on the valence band. For the treatment of organic compounds involving oxidation, both e– and h+ can react with water molecules to form oxidative reactive species (for example, OH^−^, ${\text{O}}_{2}^{-}$, and H_2_O_2_) (Fomina and Gadd, 2014). Although various publications discuss PEO, the chemicals that cause degradation in wastewater are classified as inorganic and organic pollutants. A recent study suggests that combining electrochemical oxidation and ultraradiation enhances the disinfection process by increasing the number of oxidizing products formed and activating them more effectively (de Vidales et al., 2015; Zapatero et al., 2015).

Therefore, the implementation of the photoelectrochemical oxidation (PEO) approach was effective in the treatment of cooling tower wastewater, leading to a reduction in contaminant levels. The study assessed the current density employed to reduce the chemical oxygen demand (COD) levels and investigated the activities of microbial populations that contribute to the formation of biofilms, a key factor in corrosion within cooling tower water (CTW). As a result of these findings, the oxidation process emerges as a markedly more favorable, efficient, cost-effective, environmentally friendly, and promising treatment option for the reduction or elimination of pollutants from wastewater.

## Materials and methods

### Sample collection

The cooling tower wastewater (CTW) was collected from the tannery industry (Ranipet Tannery Effluent Treatment Limited) (latitude 12.9149857° and longitude 79.3459513°) located in Ranipet, Tamil Nadu, India. The samples from the outlet of the cooling tower system were collected into a sterile container and stored at 4°C for future use.

### Physicochemical parameters analysis

The collected samples were analyzed for specific parameters, such as temperature, pH, turbidity, total suspended solids (TSS), total dissolved solids (TDS), chloride (Cl^−^), hardness, conductivity, and chemical oxygen demand (COD) using the dichromate method in the Merck Spectroquant TR 320 (Swaroop et al., 2016). The assessment followed the standard protocol outlined in the American Public Health Association (APHA) 200511, using which the physicochemical parameters of the cooling tower wastewater were evaluated both before and after treatment.

### Metagenomic sequencing analysis

#### Genomic DNA extraction

GenomicDNA extraction was carried out from the collected CTW using commercially available kits such as the Xploregen kit. Before PCR amplification, the extracted DNA from the samples was subjected to NanoDrop and GEL Check. The NanoDrop readings of 260/280 at an approximate value of 1.8–2 are used to evaluate the quality of the DNA (Prakash et al., 2021).

### Amplicon generation

The PCR amplification of the V3–V4 Region of 16s Gene: TAQ Master MIX contains High-Fidelity DNA Polymerase, 0.5 mM dNTPs, 3.2 mM MgCl2, PCR Enzyme Buffer. Primer information: 16s F:-5′ AGAGTTTGATGMTGGCTCAG3′ 16s R:-5′ TTACCGCGGCMGCSGGCAC3′. The following conditions were used: For amplification, 40 ng of the extracted DNA is utilized, along with 10 pM of each primer, with initial denaturation at 95°C, followed by 25 cycles of the following conditions: denaturation at 95°C for 15 s, annealing at 60°C for 15 s, elongation at 72°C for 2 min, and final extension at 72°C for 10 min, followed by a hold at 4°C. GEL Check and Nanodrop QC are performed on the amplified 16s PCR Product. The NanoDrop readings of 260/280 at an approximate value of 1.8–2 are used to evaluate the quality of the DNA (Shukla et al., 2021).

#### Library clean-up and sequencing

The amplicons from each sample were purified with Ampure beads to eliminate unneeded primers, and an additional eight cycles of PCR were performed using Illumina barcoded adapters to produce the sequencing libraries. Ampure beads were used to purify the libraries, and the Qubit dsDNA High Sensitivity assay kit was used to quantify them. Illumina Miseq with a 2x300PE v3–v4 sequencing kit was used for sequencing (Parthipan et al., 2023).

### Bioinformatics analysis

NCBI is the database utilized for the 16s V3–V4 region. The sequencer's bcl data is de-multiplexed and converted into fastq raw data. The quality of the de-multiplexed data will be evaluated using the Fastqc (Version 0.11.9) and Multiqc (Version 1.10.1) tools. The QC-passed samples are qualified for further analysis, and for 16s metagenomics, we employed our own pipeline (Biokart Pipeline). The pipeline procedure is as follows: quality control, chimaera identification, OIU clustering, choosing representative sequence, assigning taxonomy, and creating a taxonomy table. When the run is finished, we have the final raw OTU table from which we can begin visualizing the results. Microsoft Excel is used to create the abundance feature tables and the top 10 organisms in each sample (2010). A microbiome analyst was used for additional studies such as a heat map, a core microbiome, a dendrogram, an alpha diversity index, a beta diversity index, a PCOA plot, a rarefaction curve, and so on (Chong et al., 2020; Mater et al., 2023). The metagenomic report is submitted to NCBI under the accession number PRJNA836405 (https://www.ncbi.nlm.nih.gov/bioproject/PRJNA836405).

### Electrolytic system

The experimental setup involved bulk electrolysis utilizing a small, open, cylindrical, and hollow-shaped electrochemical tubular flow cell coated with a titanium tube. This tube had a diameter of 21 cm, a length of 3.9 cm, and a volume of 370 mL. The anode and cathode were positioned with a distance of 1.5 cm between them, and the cathode chamber was equipped with outlets and inlets for electrolyte flow. The inner side of the titanium tube housed the titanium-coated (Ti/TiO2-RuO2) expanded anode mesh, while the spacing between the cathode and anode was ~3.2 cm in length, with a diameter of 1 cm. The electrodes were arranged in parallel and vertically, maintaining an inter-electrode gap of 4 cm. The electrolyte sample, comprising 2,500 mL of CTW, was circulated through a peristaltic pump at a flow rate of 440 mL/min, and a DC supply was applied at a current density of about 12.21 mA/cm^2^. This setup was employed for various electrochemical studies, following the methodologies outlined in previous studies (Su et al., 2019; Baaloudj et al., 2023). In the following studies, the three CTW systems were subjected to three treatments to test the comparative efficiency of the treatment process: (i) electro-oxidation (EO), (ii) photo-oxidation (PO), and (iii) photoelectro oxidation (PEO).

In the EO treatment (S2), current was applied to the electrolyte solution to treat the CTW (Martínez-Huitle and Panizza, 2018). In the PO (S3) setup, the UV lamp was only used to treat the CTW (Marszałek and Puszczało, 2020; Yi et al., 2022). In the PEO (S4) process, the rod-shaped UV lamp (6W) (Heber) was inserted in the middle of the anode mesh (Sierra-Sánchez et al., 2022) as the control system without treatment (S1). The total working volume flowed through the peristaltic pump to prevent temperature rise (Elawwad et al., 2017). The experiments were conducted under applied current conditions, by providing a direct DC supply (Aplab LD 6402). The entire experiment extended over 6 h, and samples were collected at 2-h intervals. The treated CTW underwent analysis post-treatment to examine COD levels and microbial growth to ensure adequate COD removal (Sundaravadivel and Vigneswaran, 2001). The processed water was further exposed to sunlight to eliminate any residual contaminants present in the CTW.

#### Electro-oxidation

In this setup process, a circulating chamber is filled with the cooling tower water to a capacity of 2,500 mL. The electrolytic solution received an applied current of 1 A. On the anodic side, oxidation reaction takes place by breaking down the internal chemicals with the required current (Martínez-Huitle and Panizza, 2018).

#### Photo-oxidation

In this process, the sample is recirculated throughout the circulating chamber without using any applied current. The UV lamp is used in this setup to remove the organic contaminants and microbes present in the cooling tower system. The total viable bacterial counts and COD were estimated at the end of the experiment (Marszałek and Puszczało, 2020; Yi et al., 2022).

#### Photoelectro-oxidation

In this system, the electro-oxidation process and UV lamps were used in parallel. The UV and external current are applied in a sample time to achieve the removal of disinfectant in the cooling tower water (Sierra-Sánchez et al., 2022).

#### Ultraviolet–visible spectroscopy

The UV–visible spectrophotometer (UV-1800 SHIMADZU, Japan) was used to determine contaminants in the water sample both before and after treatment. APHA color measurements were quantified using the Hazen color index, and COD analysis was done at time intervals of 0, 2, 4, 6, and 8 h (Prakash et al., 2021).

### Biocorrosion study

#### Weight loss measurement

Following the methodology outlined by Kokilaramani et al. (2021), the weighted samples in the form of coupons (2.5 × 2.5 cm^2^) of the MS 1010 were introduced into the electro-oxidation (EO), photo-oxidation (PO), and photoelectro-oxidation (PEO) systems. The total number of corrosion formation systems was 4 (*n* = 4). Preweighted metal coupons, with each system having three coupons, were positioned in 500 mL conical flasks containing 400 mL of either pretreated or treated CTWs. These systems included S1—control (cooling tower water), S2—electro-oxidation-treated cooling tower water, S3—photo-oxidation-treated cooling tower water, and S4—photoelectro-oxidation treated cooling tower water, each in triplicate with an immobile condition. After 14 days of exposure, the coupons were retrieved from the flask, and non-metallic scrapers were employed to remove the corrosion products.

Following the completion of incubation, samples were taken from system and washed with pickling solutions. They were then dried at 40°C until they reached the desired weight consistency and then weighed again; the weight disparities were observed. The corrosion rate of MS was then analyzed and reported in mils per year (mpy), adhering to ASTM G1 standards. The weight loss calculation was performed to determine the corrosion rate (CR) using standard formulas and statistical significance.

#### Fourier-transformed infrared spectroscopy analysis

After the weight loss experiment, FT-IR was used to analyze the metal-scrap powdered samples. The FT-IR spectra with a wave number range of 400–4,000 cm^−1^, a resolution of 8 cm^−1^, and a scan rate of 64 scans/spectrum (PerkinElmer Spectrum IR Version 10.6.0, USA) were employed. Potassium bromide (KBr) was used to make pellets by applying hydrolytic pressure on metal samples (Gebreslassie et al., 2019). FT-IR was used to analyze the compounds present in the sample.

#### Scanning electron microscopy analysis

The surface morphologies and elemental compositions of mild steel samples exposed to pretreated and treated CTW for 21 days were examined by SEM/EDX by a Tescan VEGA 3SBH with an EDX detector on high magnification and resolution environmental scanning electron microscope (Morsi et al., 2016). After 21 days of exposure, the specimen surfaces S1, S2, S3, S5, S7, S9, and S11 were examined in order to assess the surface microstructure and composition of biofilm formation (Gebreslassie et al., 2019). To remove loosely adsorbed ions, the mild steel samples were finally washed thoroughly and then subjected to 15 min of ultrasonic cleaning.

#### X-ray diffraction studies

The XRD on metal powder, which had been previously prepared in a mortar, was analyzed by Bruker D8 Advance Equipment (Germany) with a LynxEye& Scintillation Counter detector with a 5°-140° angular range and at a rating between 40 and 30 kV. XRD analysis was applied directly to the mild steel samples in order to determine the types of oxide layers and corrosion products present on their surfaces (Narenkumar et al., 2019).

#### Electrochemical studies

The electrochemical analysis, including impedance spectroscopy and polarization, was conducted using the Metrohm Autolab instrument, USA, accompanied by Nova 2.1.5 software. This conventional three-electrode system has a capacity of ~50 mL of electrolyte, with MS specimens serving as the working electrode, platinum (Pt) as the counter electrode, and a saturated calomel electrode (SCE) as the reference electrode (Yue et al., 2014). After a 30-min immersion in the test fluid with a signal amplitude perturbation of 5 mV, the working electrode achieved a steady-state open circuit potential, *E*_corr_. Impedance and potentiodynamic studies were performed on MS 1010 coupons (4 nos.) acting as the working electrode, with the reference electrode being a saturated calomel electrode (SCE) and a platinum electrode as the counter electrode (Mayrhofer et al., 2009). Impedance measurements were conducted through an AC signal output with an amplitude of 10 mV at an open circuit potential (OCP) within the frequency range of 0.1–100 kHz. The impedance data were fitted to the most appropriate equivalent circuit, and the impedance parameters were obtained from Nyquist plots. The double-layer capacitance (Cdl) values were calculated from the frequency at the imaginary component of impedance (Grassini et al., 2018). The results were obtained 10 min after the startup process to reach a constant potential and were connected with multiple electrodes at a scan rate of 1,800 mV/h from an open circuit potential of +200 mV SCE to −200 mV SCE. The cooling tower water sample was analyzed after 21 days of immersion.

## Results and discussion

### Physicochemical characterization of cooling tower wastewater due to electrochemical oxidation

The physicochemical parameters of pretreated and treated CTW in EO, PO, and PEO are presented in Table 1. The pH of the CTW ranges from 5.71 to 4.28, having an average value of 5.70, which is permissible. The wastewater's turbidity exhibits a pale or dark yellow color, with a color unit of 0.48 Hazen. As the permissible limit of color according to the Central Pollution Control Board (CPCB) is 10 Hazen, the wastewater is treated to reduce the color before discharge (Srivastava et al., 2016). The average values of total suspended solids (TSS) and total dissolved solids (TDS) are found to be 133 ± 4 and 225 ± 0.5 mg/L, respectively. The chloride content in wastewater is determined to be 367.20 ± 3 mg/L, which is below the acceptable threshold of 1,000 mg/L. Additionally, the average value of conductivity is 1,348 ± 0.2 μS/cm, and the wastewater has chemical oxygen demand (COD) values (average) of 185 ± 6 mg/L. These results indicate that PEO treatment (2,512 ± 3 to 185 ± 6 mg/L) effectively reduced the COD values (Martínez-Huitle and Panizza, 2018). This result is in agreement with the data previously reported by Jallouli et al. (2020) for COD reduction in wastewater electrocoagulation (EC) or UV photolysis, which achieved 85.7 and 55.9% of COD reduction, respectively. Our results also broadly agree with these values. The average values of sulfide and sulfate are 112 ± 3.9 and 123 ± 2.1 mg/L, respectively, while the values of hardness and alkalinity are 170 ± 1 and 212 ± 1.5 mg/L, respectively. The COD test is crucial for detecting hazardous circumstances, biologically resistant organic substances, and the performance of treatment facilities due to the rapid results it provides. PEO emerges as the most efficient approach, as evidenced by the results of the electrochemical treatments. Thus, the efficiency of the methods is of the order PEO > EO > PO.

**Table 1:** Estimation of chemical parameters of cooling tower water.

**Test parameter**	**Unit**	**Control**	**EO**	**PO**	**PEO**
Temperature	°C	28 ± 0.1	35 ± 0.3	35 ± 0.5	35 ± 0.1
pH	1–14	5.71 ± 0.1	4.28 ± 0.3	4.93 ± 0.2	5.70 ± 0.6
Turbidity	NTU	5.09 ± 0.6	1.97 ± 0.1	2.13 ± 0.5	0.48 ± 0.2
TSS	mg/L	1,000 ± 5	202 ± 6	245 ± 2	133 ± 4
TDS	mg/L	700 ± 0.8	626 ± 0.6	689 ± 1.2	225 ± 0.5
Cl^−^	mg/L	634.55 ± 6	570.74 ± 4	616.83 ± 7	367.20 ± 3
Conductivity	μS/cm	2,515 ± 0.4	2,076 ± 0.2	2,394 ± 0.3	1,348 ± 0.2
COD	mg/L	2,512 ± 3	1,380 ± 3	1,428 ± 5	185 ± 6
Sulfide	mg/L	888 ± 2.0	412 ± 3.3	682 ± 3.5	112 ± 3.9
Sulfate	mg/L	1,957 ± 1.0	582 ± 1.1	1,724 ± 3.3	123 ± 2.1
Hardness as CaCO3	mg/L	1,250 ± 2	1,120 ± 2	1,175 ± 3	170 ± 1
Alkalinity	mg/L	1,746 ± 1.3	741 ± 1.4	1,000 ± 2.2	212 ± 1.5

EO, electro-oxidation; PO, photo-oxidation; PEO, photoelectrochemical oxidation.

### Metagenomic analysis

#### Isolation, characterization, and next-generation (metagenomic) sequencing of microbes

**Figure 1:**
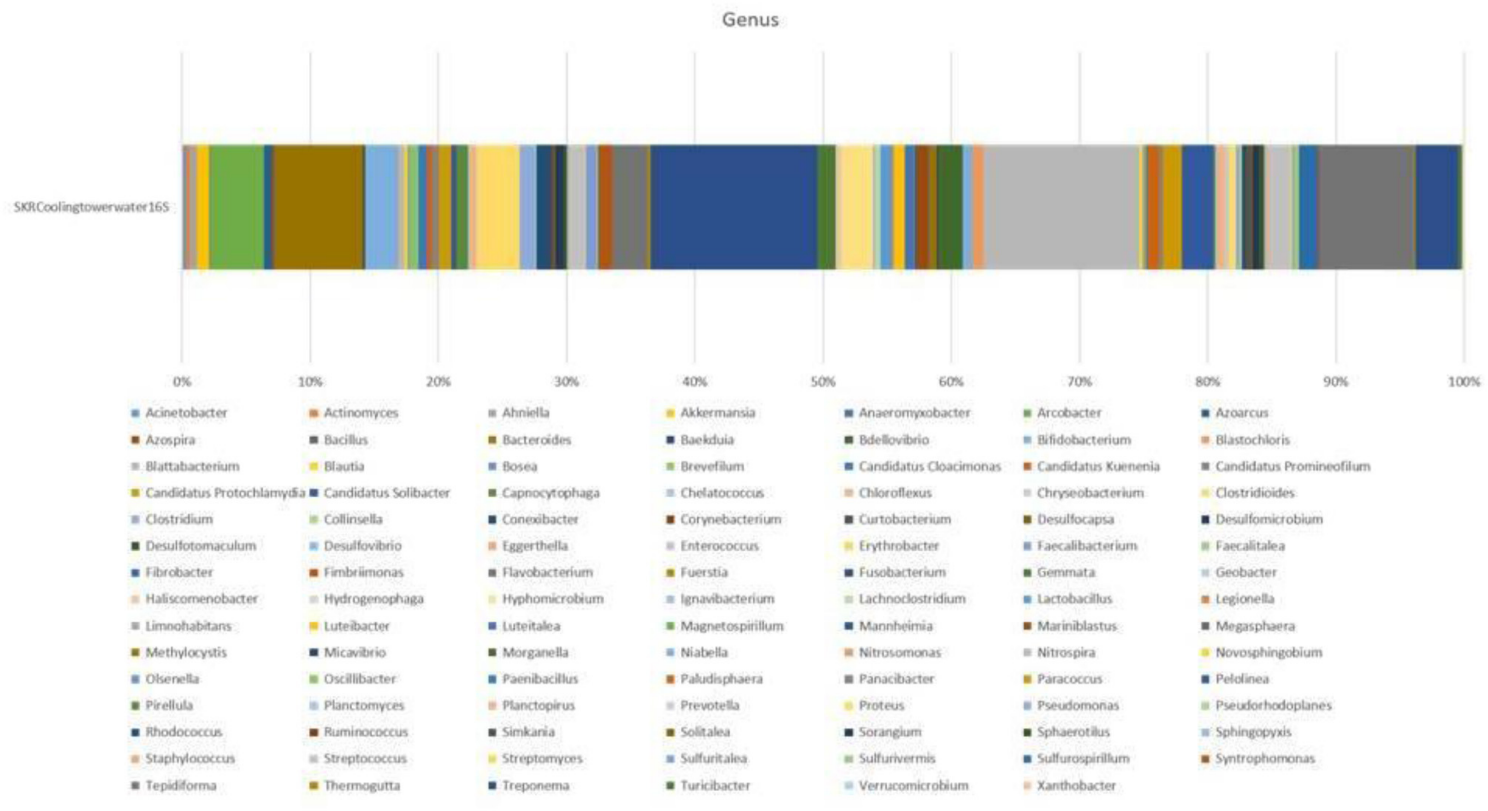
Genus revealed by metagenomics studies in the collective sample of cooling tower wastewater.

The taxonomic annotation of the predicted operational taxonomic units (OTUs) showed that CTW metagenomics was dominated by the domain bacteria (77.50%), followed by unknown (12.50%), and <10% OTUs were observed for archaea, viruses, and eukaryotes (Petrosino et al., 2009). Microbial diversity was accomplished in detail through taxonomic classification, which was performed using a ribosomal database project (RDP) classifier against the SILVA operational taxonomic units (OTUs) database. The detailed information of the CTW sample including total consensus reads chimeric sequences, pre-processed reads, OTUs, phylum (Supplementary Figure S1), class (Supplementary Figure S2), order (Supplementary Figure S3), family (Supplementary Figure S4), genus (Figure 1), and distribution for each sample based on OTUs and reads (Supplementary Figure S5) The findings of the metagenomic study were included in a submission to NCBI, with BioProject Accession number PRJNA836405 (https://www.ncbi.nlm.nih.gov/bioproject/PRJNA836405). It was noted that the taxa outside of top most 10 taxas are classified. At the genus level of taxonomic profiling, a total of 105 bacterial genera were detected in the CTW (Raji, 2017). Particularly when contrasted with variances within the individual treatments, the differences between the communities in the cooling tower systems, both in the planktonic cells and in the biofilm developed over the coupons, were significant. When the OTU numbers was investigated, *Fusobacteriia* class members predominated in planktonic communities (Gambino et al., 2022). The water sample consists of the most abundant bacterial OTU class (%) as follows (Figure 1): *Fusobacterium* (22), *Nitrospria* (21), *Tepidiforma* (13), *Bacteroides* (12), *Arcobacter* (7), *Clostridioides* (6), *Treponema* (6), *Flavobacterium* (5), *Bifidobacterium* (4), and *Hyphomicrobium* (4). The water sample contains diverse microbial genera (Figure 1) such as *Corynebacterium, Actinomyces, Streptomyces, Collinsella, Olsenella, Eggerthella, Baekduia, Clostridioides, Streptococcus, Lactobacillus, Bacillus, Xanthobacter, Erythrobacter, Sulfuritalea, Acinetobacter, Sulfurivermis, Desulfovibrio, Desulfomicrobium, Desulfotomaculum, Geobacter, Bacteroides, Fibrobacter, Pseudorhodoplanes, Nitrospira, Fimbriimonas, Flavobacterium, Pseudomonas, Clostridium*, and *Staphylococcus* (Figure 1) (Prakash et al., 2021). The metabolic activates of cooling tower water in the bar graphs representing microbial community composition revealed metagenomes at taxonomic levels in the cooling tower wastewater: (a) phylum, (b) class, (c) order, (d) family, and (e) genus. Microbial taxa showing <1% relative abundance are shown as “others” in the phylum level gto family level bar graphs, whereas those <1.5% are presented in the genus level bar graph (Pinel et al., 2020).

**Figure 2:**
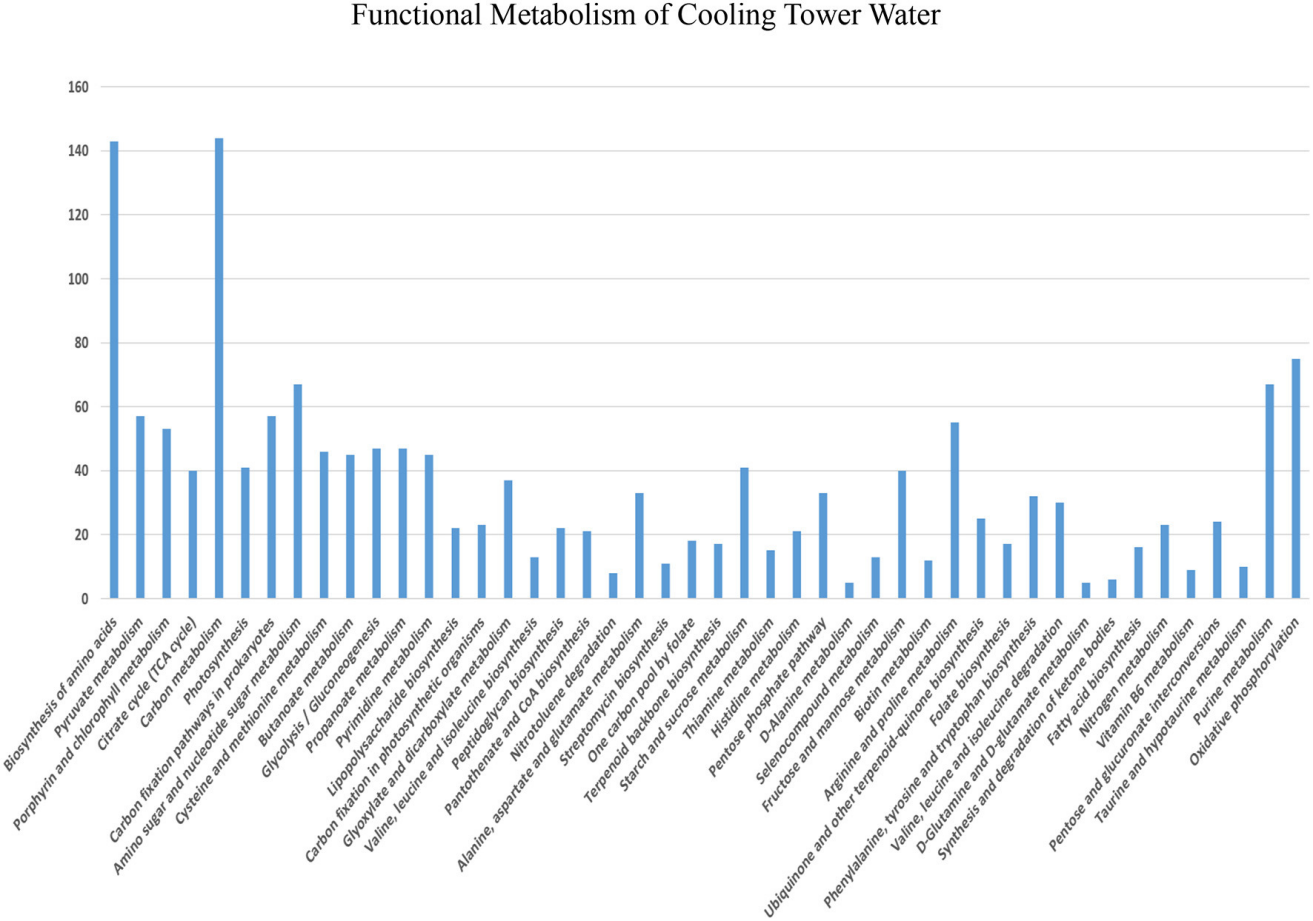
Metabolic activities in the collective sample of cooling tower wastewater.

The functional metabolism of cooling tower water is shown in Figure 2. According to the results of the CTW metabolic analysis (Supplementary Table S1), nearly 46 metabolic pathways were identified, and of these, carbon metabolism was ~308 (97.2%), biosynthesis of amino acids was 225 (71%), oxidative phosphorylation was 202 (63.8%), purine metabolism was 178 (56.2%), and arginine and proline metabolism stands at 129 (40.7%). Metabolism is a process involving the utilization of organic material for energy or the creation of cellular activities, occurring in all organic molecules to produce the acetate discharged as acetyl-CoA employed for biosynthetic reactions, which is oxidized to CO_2_ accompanied by an equivalence reduction in tricarboxylic acid (TCA) cycle (Parthipan et al., 2023). The reduction equivalency was measured in the respiration chain with oxygen molecules. In this reaction, 2 M of acetate is oxidized to yield 34 M of ATP when the electron transport chain is phosphorylated, with oxygen acting as the terminal electron acceptor. This process preserves ATP. The ATP anhydridic phosphate bond energy has values up to 2 M upon 1 M glucose glycolysis. During metabolism, reducing equivalents participate in a controlled combustion process with molecular oxygen. Methane, CO_2_, NH_3_, and H_2_S are produced by fermentative and acetogenic bacteria in conjunction with methanogens or sulfate reducers. It takes a low hydrogen partial pressure, mostly from interspecies hydrogen transfer, for biopolymers or monomers to completely degrade methanogenesis (Holtzapple et al., 2021). By arranging acetogens and hydrogenolytic methanogenic bacteria within short diffusion distances in flows or biofilms, interspecies hydrogen transfer is facilitated, and the fermentation process produces reducing equivalents for the reduction of carbon dioxide to methane and sulfate to sulfur dioxide (Gallert and Winter, 2005). The metabolism enzymes in the form of most toxic microorganisms catalyze the formation of analog chemicals, which are characterized as extracellular substances. These enzymes contribute to the mineralization of organic materials in water (Ahmad et al., 2022).

#### UV light spectrophotometric analysis

**Figure 3:**
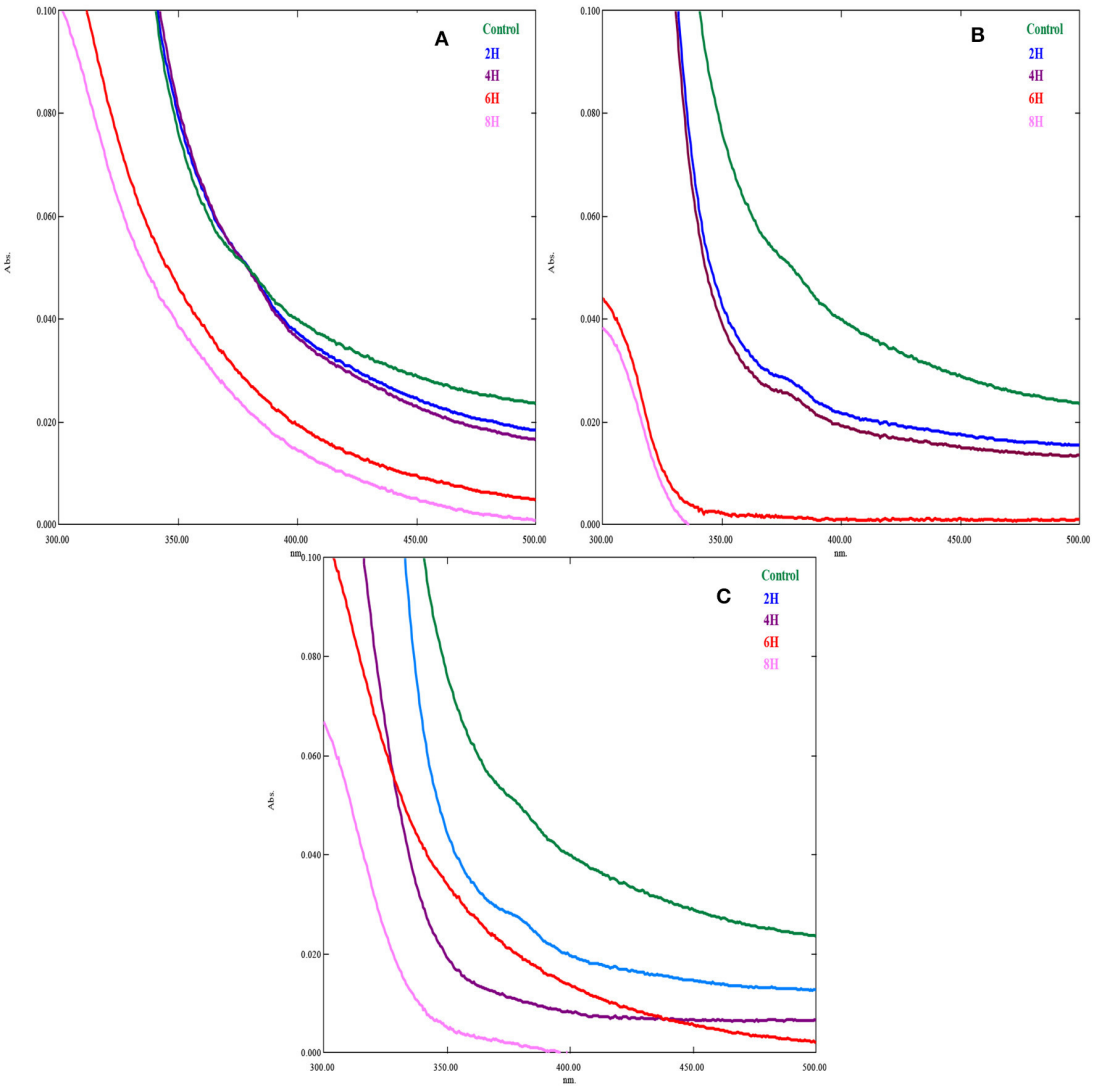
UV spectrum of treated and untreated cooling tower wastewater in different hours. **(A)** EO; **(B)** PO; **(C)** PEO.

Figures 3A–C show the UV spectrum of cooling tower wastewater at different hours following EO, PO, and PEO. The adsorption UV spectrum indicates that the oxidation of CTW varies widely between the different hours (Ahmed et al., 2022). These entire spectrums are transformed in order for it to have an identical area between 300 and 500 nm, the range of the multicomponent method. The absorption spectra of the sample were identified in the visible range. The band at nearly 350 nm is distinctive of the unique aromatic rings and shows a considerable range of aromatic ring oxidation in the molecules of the wastewater. When the wastewater was exposed to UV light, a quick oxidation reaction takes place; this reaction time was virtually consistent for the photo-oxidation of the organic components in an aqueous solution (Zheng et al., 2022). The acquisition of a UV spectrum of a sample without treatment is carried out with a UV light (Advanced UV Examination of Wastewater). The existence of organic compound(s) present in the formulation that may absorb in the UV region is the primary explanation for the differences between the UV spectra of CTW (Yi et al., 2022). The whole spectrum range is between 200 and 800 nm, and for their dissolution in complex biological and environmental matrices, the working wavelengths are 325 and 395 nm. The slope break of the UV spectrum of fraction 4, at ~240 nm, is softened, confirming the presence of large particles (Physical and Aggregate Properties Marie-Florence Thomas) (Kinetics of decolourization of the azo structure in wastewater by UV/H2O2 process).

#### Weight loss measurement

The results of weight loss of biocorrosion system in cooling tower water are presented in Table 2. Initially, the MS coupon 1010 was weighed for the biocorrosion studies. In the control system, S1—pretreated CTW weight loss was recorded as 0.109 ± 0.04 mg, S2—EO-treated CTW weight loss was 0.98 ± 0.06 mg, S3—PO-treated CTW weight loss was 0.64 ± 0.03 mg, and S4—PEO-treated CTW weight loss was 0.33 ± 0.05. The corrosion rate on exposure in S1, S2, S3, and S4 was 1.970, 0.859, 0.566, and 0.249, respectively (AlSalhi et al., 2023). After analyzing the statistical variance, it was found that the corrosion rate result, *p* < 0.05, is statistically significant.

**Table 2:** Weight loss of biocorrosion system in cooling tower water.

**Systems**	**Weight loss (mg)**	**Corrosion rate (mm/year)**
S1—control (cooling tower water)	0.109 ± 0.04	1.970
S2—electro-oxidation treated cooling tower water	0.98 ± 0.06	0.859
S3—photo-oxidation treated cooling tower water	0.64 ± 0.03	0.566
S4—photo-electro-oxidation treated cooling tower water	0.33 ± 0.05	0.249

The weight of the treated sample as a result of exposure period before the test is expressed as a ratio to its total mass. On a comparison of the pretreated and treated (EO, PO, and PEO) systems, the samples exhibiting the most severe metal loss behaviors offered superior conditions for the physical, chemical, and microbiological activities over a period of time. By using a mix of electric and UV light exposure to oxidize the microbial community and chemical substances, the PEO system exhibits negligible metal loss. The same set-equivalent corrosive conditions increased trust, and the parallel setup's identical corrosion conditions provided much more comfort (Rafieenia et al., 2022). The weight loss in the control system was also caused by the presence of inorganic chemicals and other metabolic processes.

#### FTIR analysis

**Figure 4:**
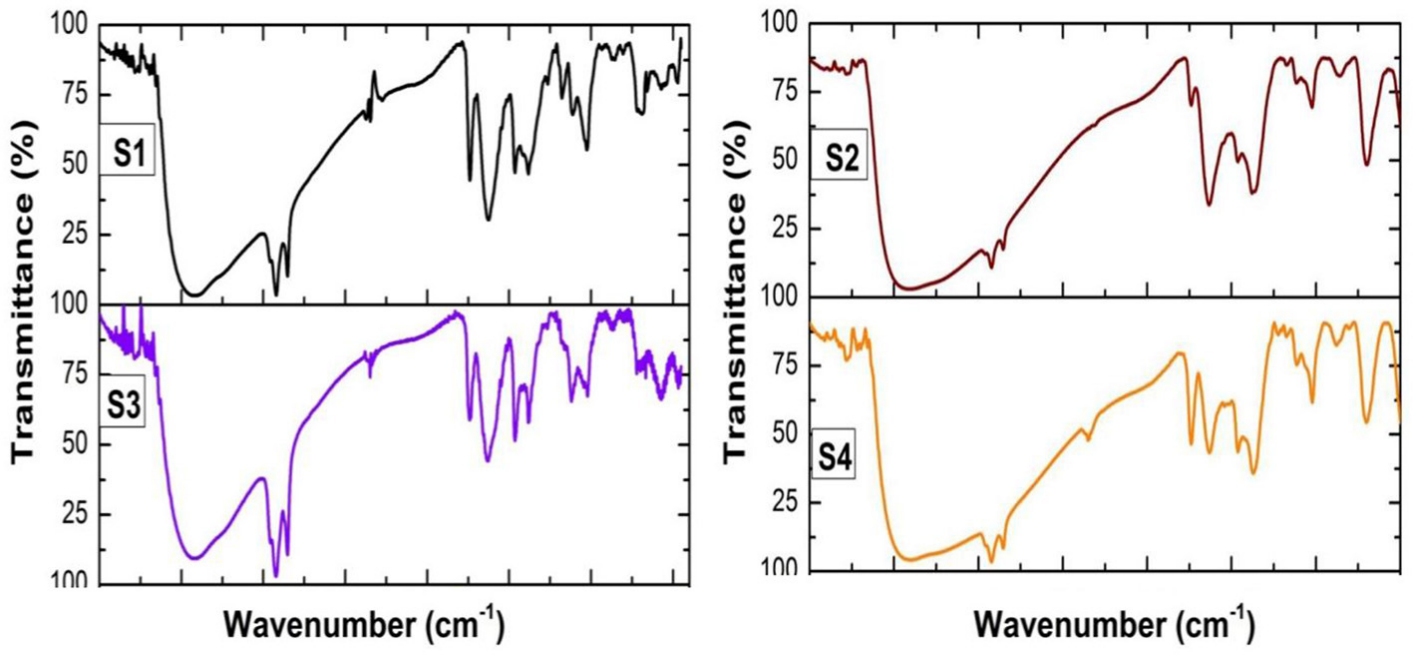
FTIR analysis of treated and untreated cooling tower wastewater. S1, Control; S2, EO; S3, PO; S4, PEO.

The IR spectra reveal potential structural changes that may have occurred during the corrosion process. Figure 4 and Table 3 present the FT-IR spectra of CTW examined before and after oxidation. The FT-IR analysis of the biocorrosion system in CTW indicates several unique bands. A qualitative and quantitative comparison of the generated peaks for both pre-treatment and treated samples demonstrates the efficacy of the contaminant removal process. The FT-IR peaks show that compounds bind with samples S1, S2, S3, and S4. The value indicates the presence of 3,423 N–H stretches, denoting wide broad, amide R-C(O)-NH-R compounds. The peak at 2,924 cm^−1^ shows the presence of strong alkenes and narrow C—H stretch peaks. The peak at 727 cm^−1^ represents aldehydes R-C-H =O, a strong COH stretch compound. The peak at 1,636 cm^−1^shows amides R-C(O)-NR′R" medium-strong C=O stretch. The peak at 1,363 cm^−1^ denotes the presence of alkenes and alkyls, medium –CH (CH3)^2^ or – (CH3)^3^ bend compound. The peak at 2,347 cm^−1^ indicates the presence of nitriles R-C = N medium strong C = N Stretch. The peak at 692 cm^−1^ shows aromatic compounds, mono-substituted strong C–H bend. The peak at 864 cm^−1^ represents alkenes RR′C=CH2 strong =C-H bend. The peak at 1,010 cm^−1^ demonstrates ethers Ar-O-R medium strong=C-O-C symmetric and asymmetric stretch. The peak at 1,109 cm^−1^ shows alcohols RR′C-OH (2°) or C=C-CRR′-OH medium strong C-O strong (Kamali et al., 2023).

**Table 3:** FTIR analysis of biocorrosion system in cooling tower water.

**Peak values (cm^−1^)**	**Bond**	**Intensity**	**Functional groups**
3,423	N-H stretch	weak-medium	Amides R-C(O)-NH-R
2,924	C—H Strech	Strong	alkanes
2,347	R-C=N	Medium strong	Nitriles
1,727	R-C-H =O	Strong	Aldehydes
1,636	R-C(O)-NR′R"	Medium strong	Amides
1,363	–CH(CH3)2 or –(CH3)3	Medium	Alkanes and alkyls
1,109	RR′C-OH (2°) or C=C-CRR′-OH	Medium strong	Alcohol
1,010	=C-O-C	Medium strong	Ethers
864	=C-H	Strong	Alkenes
692	C-H	Strong	Aromatic compounds

#### SEM analysis

**Figure 5:**
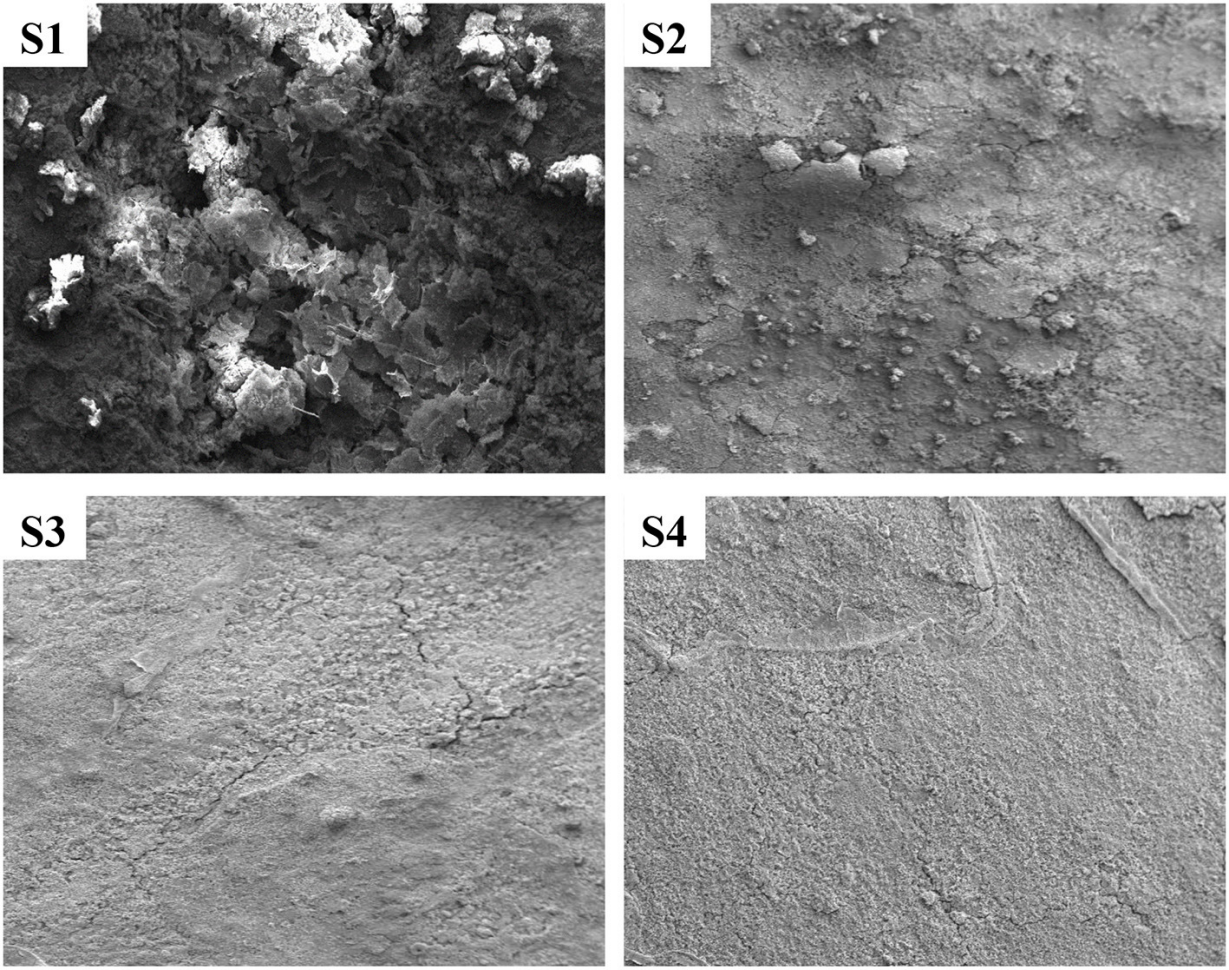
SEM analysis of treated and untreated cooling tower wastewater. S1, Control; S2, EO; S3, PO; S4, PEO.

The SEM images of the pretreated and treated CTW specimens incubated for 21 days are depicted in Figure 5. Basically, a thick layer filled the entire specimen's surface. However, as can be observed, the arrangement of deposition on the metal surface and these oxide layers do not resemble each other. The deposition covering the pretreated corroded areas exhibits a layered structure, with dense aggregate in the top layer (Figure 5; S1). Deposits that create a thick, fragile oxide layer on top of the specimens exposed to EO are also heterogeneous, plain, and heterogeneous form (Figure 5; S3). On their oxide layer, the cell bodies were not easily discernible. In Figure 5 (S4), the mild steel was coated with a thick, homogeneous, and plain layer deposit in the treating conditions (Parthipan et al., 2023).

**Figure 6:**
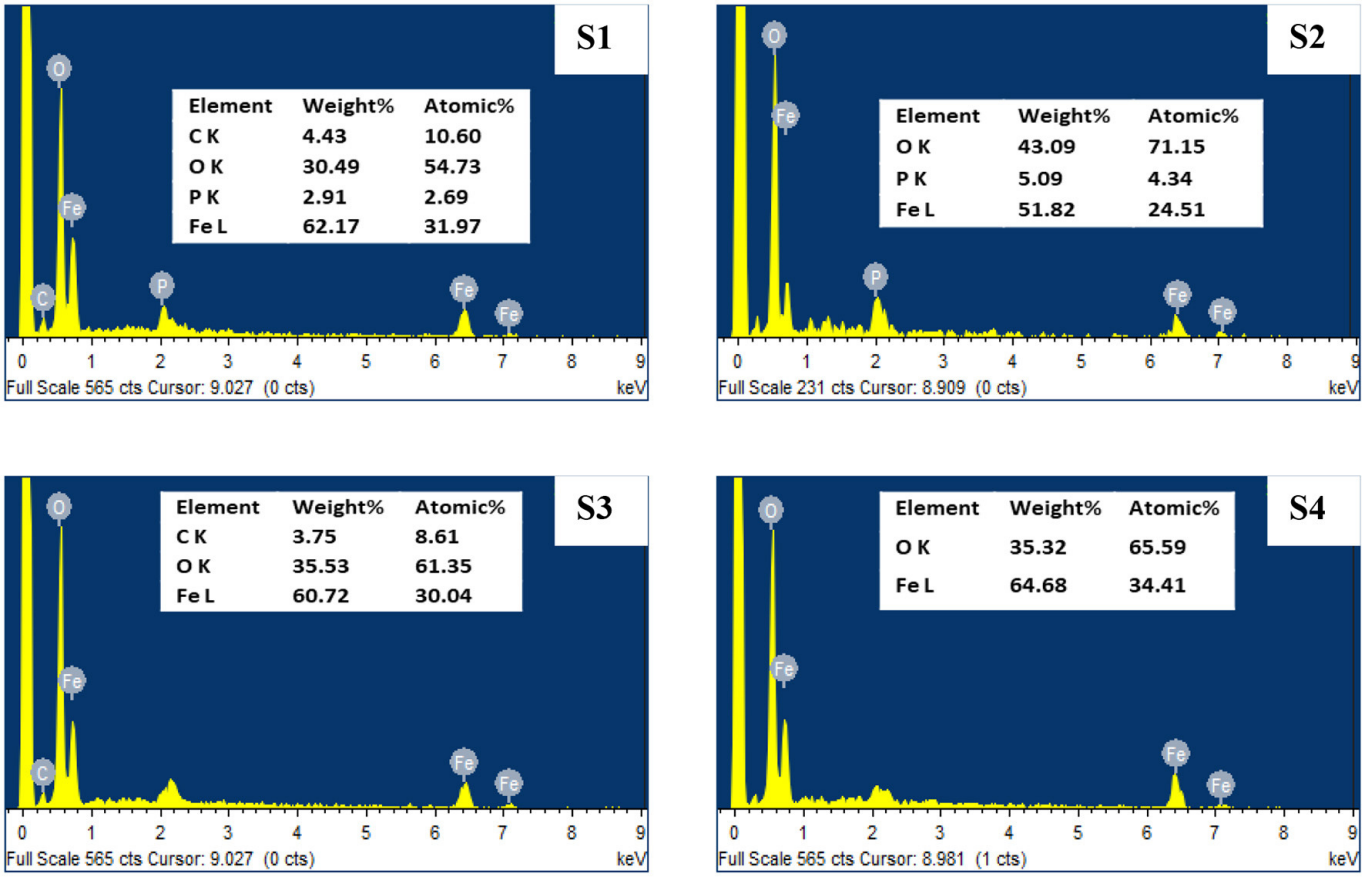
SEM analysis of treated and untreated cooling tower wastewater. S1, Control; S2, EO; S3, PO; S4, PEO.

The energy-dispersive x-ray spectroscopy (EDX) examination for mild steel surface before and after treatment with CTW (Figure 6) revealed the reduction in ions to element in the reaction mixture, which may be used to determine the existence of the elements. Strong metallic ion signals were visible in the EDX spectrum, coupled with faint oxygen and carbon peaks, which could have come from surface-bound pretreatment water sample (Rocha et al., 2014). The EDX spectra of pretreatment water samples (Figure 6; S1) indicated the typical peaks (i.e., C, O, Fe, and P signals) of the formation of biofilm on the surface. The C, P, and some Fe singles are absent from the treated water (Figure 6; S2–S4), and the strength of the O peak is reduced. It is possible that these elements were completely oxidized during the treatment process (Sierra-Sánchez et al., 2022) (inhibition activity of Seaweed extract for mild carbon steel corrosion in saline formation water).

#### XRD analysis

**Figure 7:**
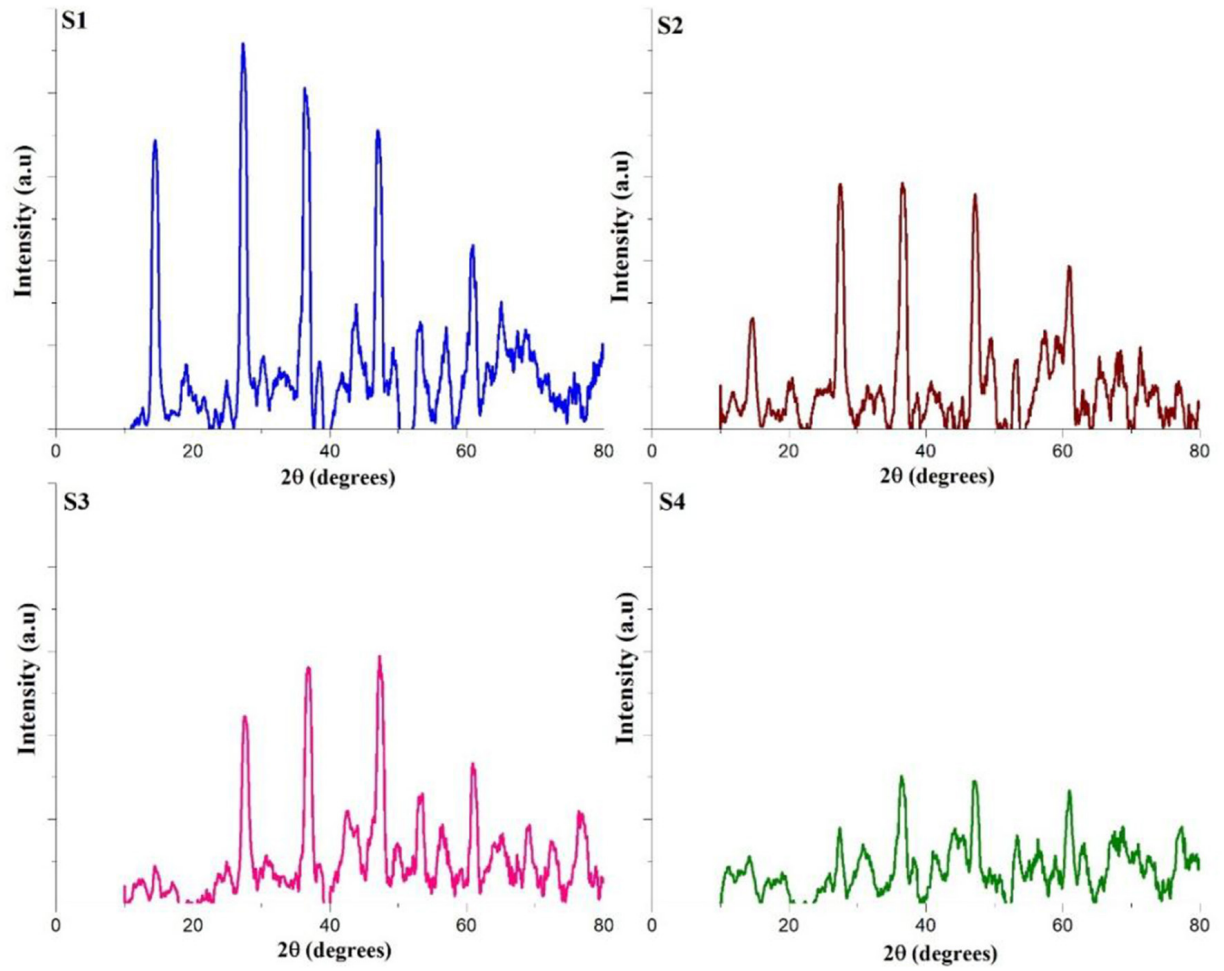
XRD analysis of treated and untreated cooling tower wastewater. S1, Control; S2, EO; S3, PO; S4, PEO.

The x-ray diffraction (XRD) pattern of corrosion products from mild steel in both pretreatment and treated CTW samples provides qualitative insights into the potential phases present (Linares-Hernández et al., 2010). The XRD data, showcasing the identified phases in the corrosion product samples, is illustrated in Figure 7. By cross-referencing and correlating the XRD data with patterns in the ICDD library, the main phase was determined to be FeOOH in all the samples. However, notable differences in peak intensity and breadth range were observed between pretreated CTW samples and treated CTW samples, indicating variations in the crystal structure (Peralta-Hernández et al., 2021). These findings suggest that the metal surfaces in the pretreatment samples had developed various types of FeOOH complexes, and the treatment process influenced the characteristics of the corrosion product phases.

#### Electrochemical studies

**Figure 8:**
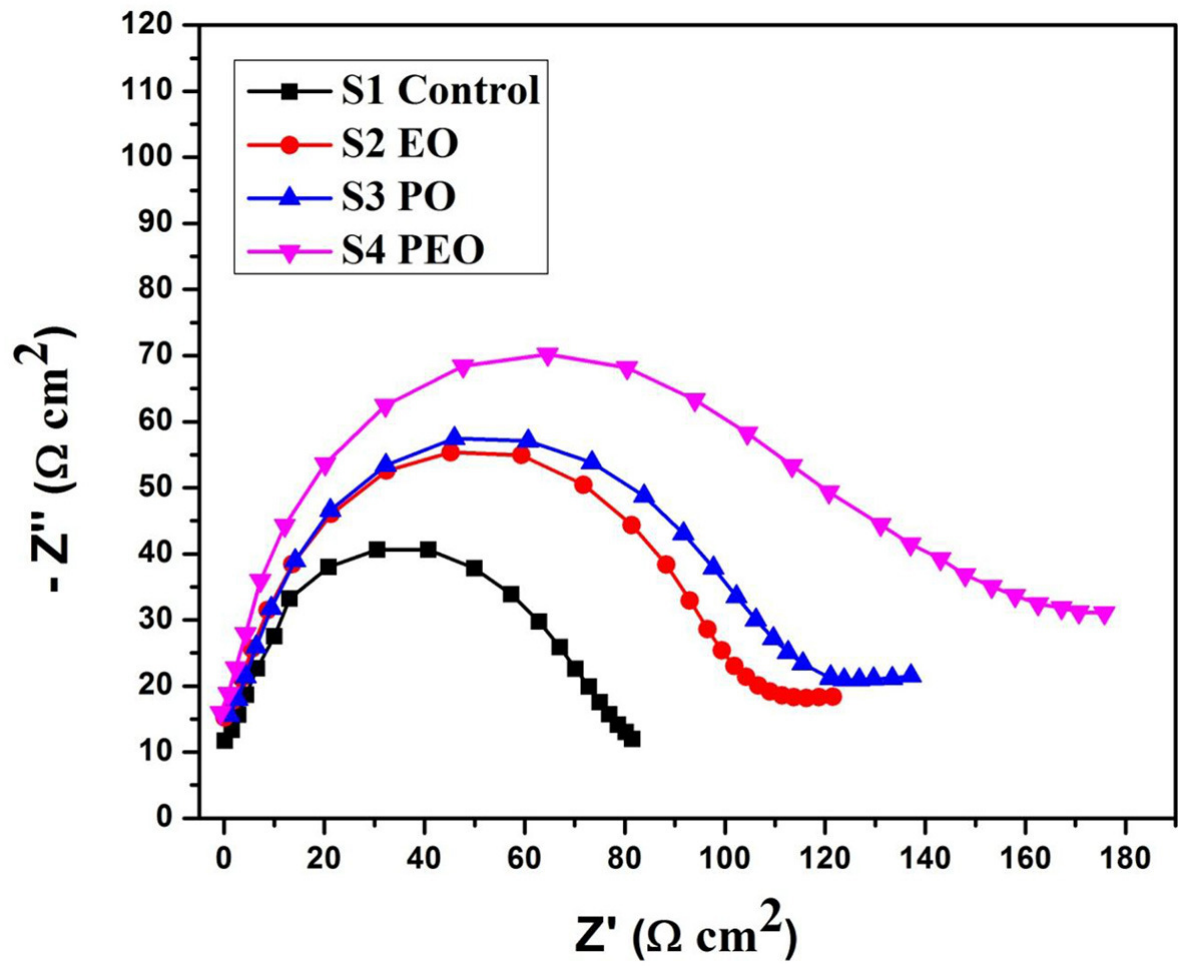
Electrochemical impendence parameters of treated and untreated cooling tower wastewater.

**Figure 9:**
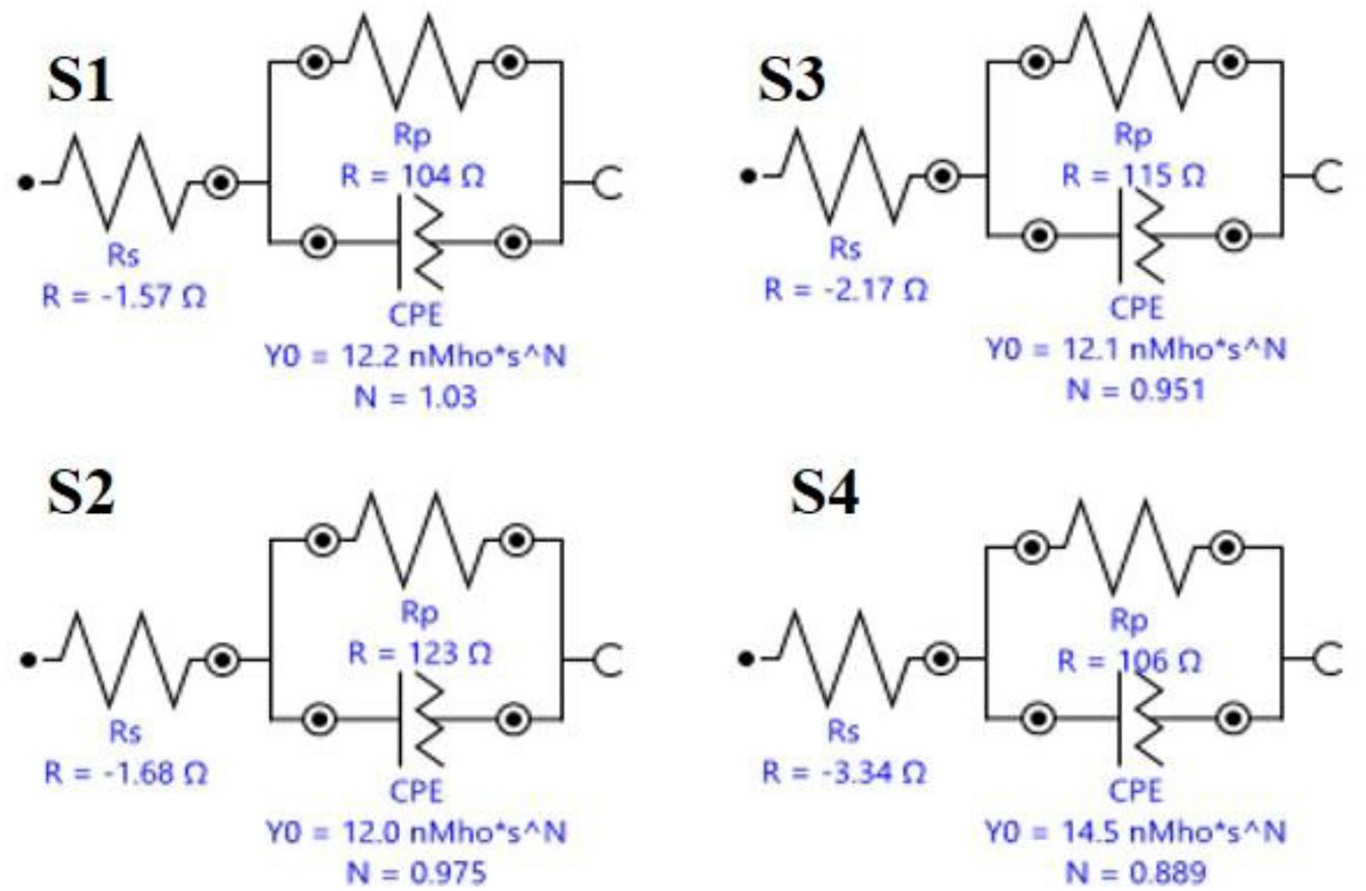
Equivalent circuits of cooling tower wastewater. S1, Control; S2, EO; S3, PO; S4, PEO.

The *I*_corr_ value of system is 8.3514E-5 for S1, 0.00018628 for S2, 7.7287E-5 for S3, and 2.8717E-5 for S4. The R_p_ value system is 582.9 for S1, 408.61 for S2, 653.81 for S3, and 557.44 for S4, as shown in Table 4 and Figure 8. Electrochemical impendence parameters of CTW were also supported the the weight loss result. The S1 control cooling tower water results exhibit black color indicating a higher impedance rate, brown color indicating greater impedance for S2, violet color compared to control for S3, and red color indicating good result for S4 (Ungureanu et al., 2020). The Nyquist plots in the presence of control were depressed semicircles at a high frequency region when compared to EO, PO, and PEO, which might be due to inhomogeneity and roughness of the metal surface (Narenkumar et al., 2021). The superior semicircles were observed in inorganic chemicals, which indicates that the treated water inhibits the formation of the barrier layer on the surface and thereby inhibits electron transfer from the metal surface. The linear polarization S1 results show black color for control, brown color indicating the higher polarization rate for S2, blue color for S3 shows the effective rate, and red color for S4 shows a good result. The EIS and potentiodynamic graphs and tables show the corrosion rate and inhibition efficiency (Table 5; Figure 9). Haaken et al., also reported that PEO gives the best results and that the latter is unfavorable because the current density constitutes a limiting electrode parameter, on the one hand, and the enhancement of the current causes an increase in the resulting voltage and thus higher energy consumption, on the other hand (Butler et al., 2011). Potentiodynamic polarization parameters of cooling tower wastewater (Table 6) represent the consistency, stability, and intensity of the passive Fe oxide film generation, and this process was carried out according the study of Rocha et al. (2014). Using both the cathodic and anodic branches of the polarization curves, Tafel plots were used to derive the current densities *I* from the polarization curves. These findings support the metal alloy sample's inclination toward corrosion resistance that was inferred from the analysis of both equivalent circuit data and EIS diagrams (Yue et al., 2014). As a result, the increase of the electric charge input should be realized by the reduction of the flow rate.

**Table 4:** Electrochemical impendence parameters of biocorrosion system in coolingtowerwater.

**Sample name**	**CEP.Y0 (F)**	**R_s_ (Ωcm^2^)**	**R_ct_ (Ωcm^2^)**	**CEP.N**
S1	1.2189E-8	−1.5714	102.43	1.0254
S2	1.1962E-8	−1.6794	121.32	0.9746
S3	1.2121E-8	−2.1749	112.83	0.95084
S4	1.4534E-8	−3.3364	102.66	0.88889

**Table 5:** Equivalent circuits for biocorrosion system in cooling tower water.

**Element**	**Parameter**	**Value**
**S1**		
R_s_	R	−1.5714
R_p_	R	104.45
CPE	Y0	1.2189E-08
		1.0254
**S2**		
R_s_	R	−1.6794
CPE	Y0	1.1962E-08
	N	0.9746
R_p_	R	122.88
**S3**		
R_s_	R	−2.1749
R_p_	R	114.94
CPE	Y0	1.2121E-08
	N	0.95084
**S4**		
R_s_	R	−3.3364
R_p_	R	106.35
CPE	Y0	1.4534E-08
	N	0.88889

**Table 6:** Potentiodynamic polarization parameters of biocorrosion system in cooling tower water.

**Sample name**	**E_corr_ (V)**	**i_corr_ (A)**	**R_p_ (Ω)**	**βa (V/dec)**	**βc (V/dec)**
S1	−0.58692	8.3514E-5	582.9	0.21065	0.23957
S2	−0.58805	0.00018628	408.61	0.44053	0.29105
S3	−0.58371	7.7287E-5	653.81	0.22714	0.23855
S4	−0.57508	2.8717E-5	557.44	0.070169	0.077651

## Conclusion

The present study highlights the significant impact of photoelectro-oxidation (PEO) in controlling microbial corrosion on mild steel in cooling tower water. The impact of bacterial culture on the corrosion of mild steel has been studied by weight loss and electrochemical parameters. This bacterium effect was suppressed by PEO-treated cooling water compared to control, EO, and PO systems. FT-IR and COD tests confirmed that PEO is an efficient method to control corrosion. Ultraviolet (UV) light, a disinfection method employed in wastewater effluent treatment, was highlighted for its ability to destroy disease-causing organisms by disrupting their genetic material, preventing reproduction. The measurement of total suspended solids (TSS), including coarse fractions such as supracolloids and settleable matter, is crucial for the characterization of water and wastewater quality. The polarization study supported the observation that the corrosion current was lower in the PEO-treated system compared to control, EO, and PO systems. The electrochemical generation of sodium hypochlorite played a vital role in destroying microbial communities, subsequently reducing the corrosion rate of mild steel in the cooling tower water system.

## Data availability statement

The datasets presented in this study can be found in online repositories. The names of the repository/repositories and accession number(s) can be found in the article/Supplementary material.

## Author contributions

SK: Writing—original draft. AS: Methodology, Formal analysis, Writing—original draft. MN: Formal analysis, Methodology, Writing—review & editing. JN: Validation, Writing—original draft. MA: Software, Validation, Writing—review & editing. SD: Investigation, Validation, Writing—review & editing. PN: Validation, Writing—review & editing. RR: Validation, Visualization, Writing—review & editing. AR: Project administration, Supervision, Validation, Writing—review & editing. TM: Validation, Writing—review & editing.
