# Supplementary material for: Application of photoelectrochemical oxidation of wastewater used in the cooling tower water and its influence on microbial corrosion
Source: Front Microbiol. 2024 Mar 13;15:1297721. doi: 10.3389/fmicb.2024.1297721 (PMC10968893; doi:10.3389/fmicb.2024.1297721)
Supplement: Supplementary file 1 [file Data_Sheet_1.docx]

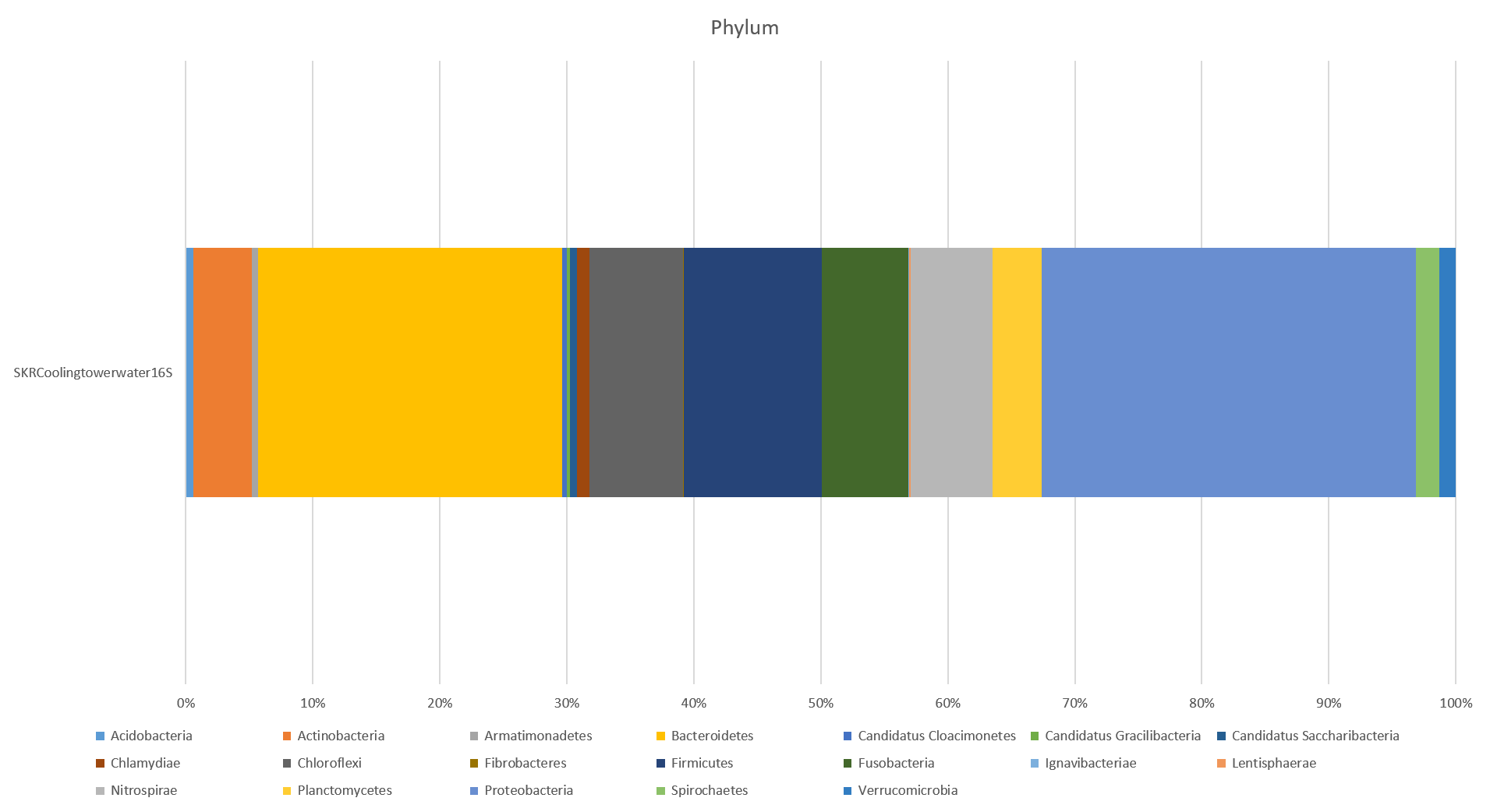
**Supplementary Document**

**SF 1. Phylum at metagenomics in the collective sample of Cooling Tower Wastewater**


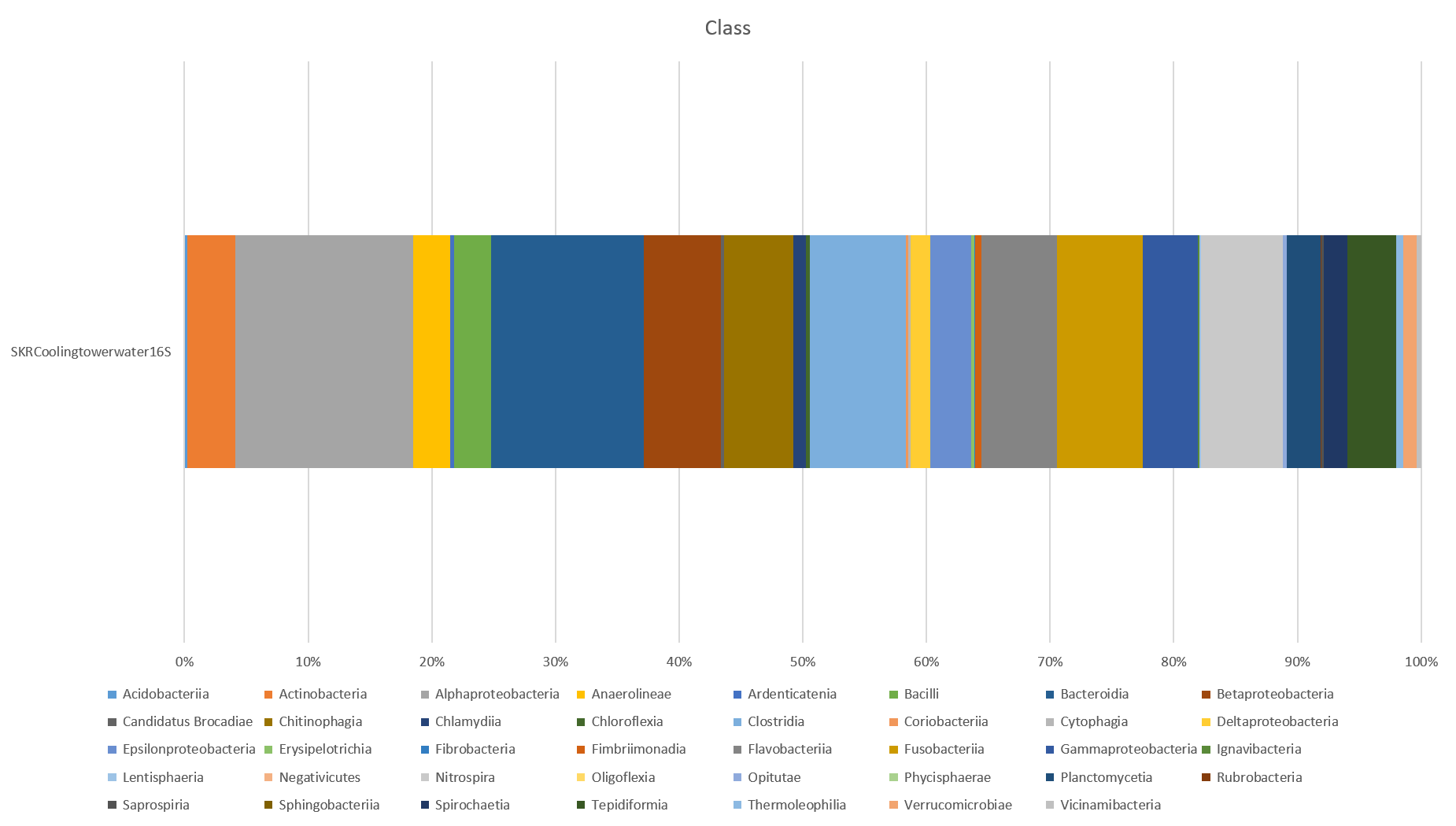


**SF 2. Class at metagenomics in the collective sample of Cooling Tower Wastewater**


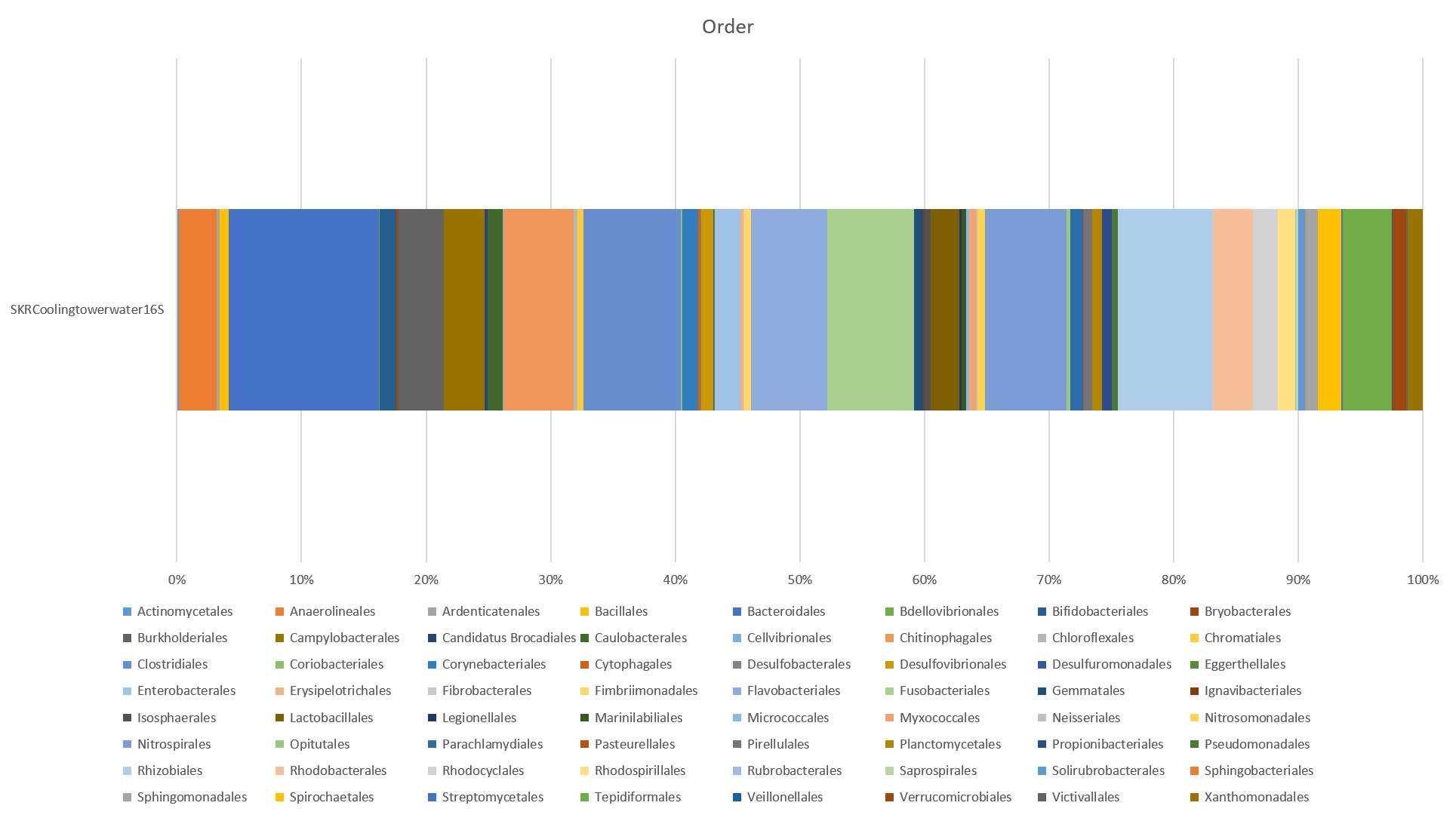


**SF 3. Order at metagenomics in the collective sample of Cooling Tower Wastewater**


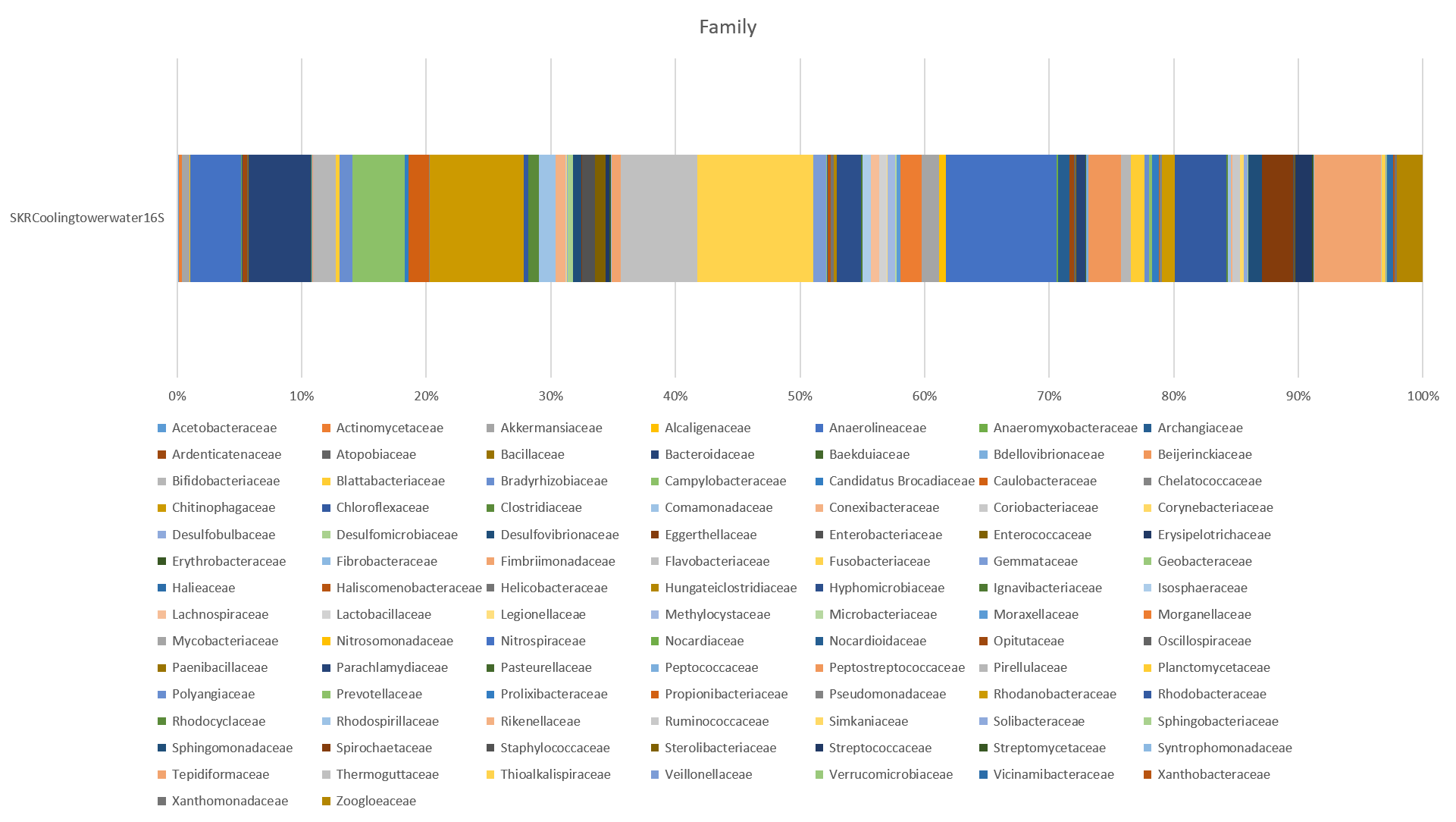


**SF 4. Family at metagenomics in the collective sample of Cooling Tower Wastewater**


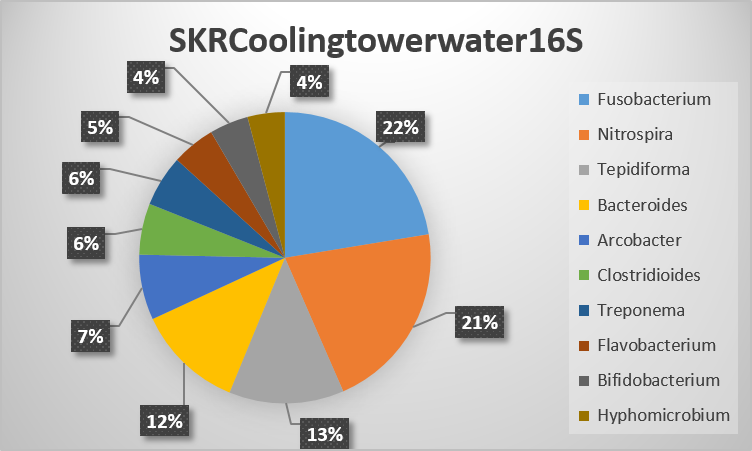
**SF 5. Top 10 Genus at metagenomics in the collective sample of Cooling Tower Wastewater**

**ST 1.Metabolic Pathway of Cooling Tower Water**

| Pathway | Total | Expected | Hits | Pval | FDR |
| --- | --- | --- | --- | --- | --- |
| Carbon metabolism | 308 | 97.2 | 144 | 3.81E-09 | 1.21E-07 |
| Biosynthesis of amino acids | 225 | 71 | 143 | 1.79E-24 | 2.84E-22 |
| Oxidative phosphorylation | 202 | 63.8 | 75 | 0.0482 | 0.167 |
| Purine metabolism | 178 | 56.2 | 67 | 0.0452 | 0.16 |
| Arginine and proline metabolism | 129 | 40.7 | 55 | 0.00456 | 0.022 |
| Amino sugar and nucleotide sugar metabolism | 125 | 39.4 | 67 | 1.66E-07 | 3.29E-06 |
| Carbon fixation pathways in prokaryotes | 101 | 31.9 | 57 | 1.39E-07 | 3.17E-06 |
| Fructose and mannose metabolism | 88 | 27.8 | 40 | 0.00393 | 0.0202 |
| Pyruvate metabolism | 87 | 27.5 | 57 | 4.64E-11 | 3.69E-09 |
| Starch and sucrose metabolism | 87 | 27.5 | 41 | 0.00152 | 0.00968 |
| Propanoate metabolism | 86 | 27.1 | 47 | 6.05E-06 | 8.01E-05 |
| Pyrimidine metabolism | 84 | 26.5 | 45 | 1.90E-05 | 0.000233 |
| Glycolysis / Gluconeogenesis | 83 | 26.2 | 47 | 1.57E-06 | 2.28E-05 |
| Porphyrin and chlorophyll metabolism | 81 | 25.6 | 53 | 2.46E-10 | 1.30E-08 |
| Cysteine and methionine metabolism | 79 | 24.9 | 46 | 6.84E-07 | 1.21E-05 |
| Butanoate metabolism | 77 | 24.3 | 45 | 7.85E-07 | 1.25E-05 |
| Phenylalanine, tyrosine and tryptophan biosynthesis | 70 | 22.1 | 32 | 0.00851 | 0.0376 |
| Glyoxylate and dicarboxylate metabolism | 69 | 21.8 | 37 | 0.000102 | 0.00102 |
| Pentose phosphate pathway | 68 | 21.5 | 33 | 0.00236 | 0.0134 |
| Alanine, aspartate and glutamate metabolism | 65 | 20.5 | 33 | 0.000888 | 0.00672 |
| Valine, leucine and isoleucine degradation | 65 | 20.5 | 30 | 0.00908 | 0.039 |
| Photosynthesis | 63 | 19.9 | 41 | 3.58E-08 | 9.48E-07 |
| Citrate cycle (TCA cycle) | 57 | 18 | 40 | 1.83E-09 | 7.29E-08 |
| Pentose and glucuronate interconversions | 55 | 17.4 | 24 | 0.0387 | 0.143 |
| Ubiquinone and other terpenoid-quinone biosynthesis | 50 | 15.8 | 25 | 0.00475 | 0.0222 |
| Nitrogen metabolism | 49 | 15.5 | 23 | 0.0167 | 0.0649 |
| Histidine metabolism | 38 | 12 | 21 | 0.00199 | 0.0117 |
| Carbon fixation in photosynthetic organisms | 36 | 11.4 | 23 | 6.10E-05 | 0.000646 |
| Peptidoglycan biosynthesis | 36 | 11.4 | 22 | 0.000229 | 0.00202 |
| Pantothenate and CoA biosynthesis | 34 | 10.7 | 21 | 0.000258 | 0.00216 |
| Lipopolysaccharide biosynthesis | 33 | 10.4 | 22 | 3.34E-05 | 0.000379 |
| Folate biosynthesis | 31 | 9.78 | 17 | 0.00587 | 0.0267 |
| Fatty acid biosynthesis | 31 | 9.78 | 16 | 0.0155 | 0.0617 |
| One carbon pool by folate | 30 | 9.47 | 18 | 0.00117 | 0.00806 |
| Terpenoid backbone biosynthesis | 28 | 8.84 | 17 | 0.00133 | 0.0088 |
| Thiamine metabolism | 24 | 7.57 | 15 | 0.0017 | 0.0104 |
| Selenocompound metabolism | 21 | 6.63 | 13 | 0.00392 | 0.0202 |
| Biotin metabolism | 19 | 6 | 12 | 0.00442 | 0.022 |
| Taurine and hypotaurine metabolism | 19 | 6 | 10 | 0.0452 | 0.16 |
| Valine, leucine and isoleucine biosynthesis | 17 | 5.37 | 13 | 0.000178 | 0.00166 |
| Streptomycin biosynthesis | 15 | 4.73 | 11 | 0.00106 | 0.00766 |
| Vitamin B6 metabolism | 15 | 4.73 | 9 | 0.0213 | 0.0804 |
| Nitrotoluene degradation | 9 | 2.84 | 8 | 0.000628 | 0.00499 |
| Synthesis and degradation of ketone bodies | 8 | 2.52 | 6 | 0.0147 | 0.0598 |
| D-Glutamine and D-glutamate metabolism | 6 | 1.89 | 5 | 0.0138 | 0.0576 |
| D-Alanine metabolism | 5 | 1.58 | 5 | 0.00311 | 0.0171 |
